# Supplementary material for: A genome-wide CRISPR screen identifies N-acetylglucosamine-1-phosphate transferase as a potential antiviral target for Ebola virus
Source: Nat Commun. 2019 Jan 17;10:285. doi: 10.1038/s41467-018-08135-4 (PMC6336797; doi:10.1038/s41467-018-08135-4)
Supplement: Supplementary file 1 — Supplementary Information [file 41467_2018_8135_MOESM1_ESM.pdf]

Supplementary Information

Flint *et al.*

"A genome-wide CRISPR screen identifies N-acetylglucosamine-1-phosphate transferase as a potential antiviral target for Ebola virus"

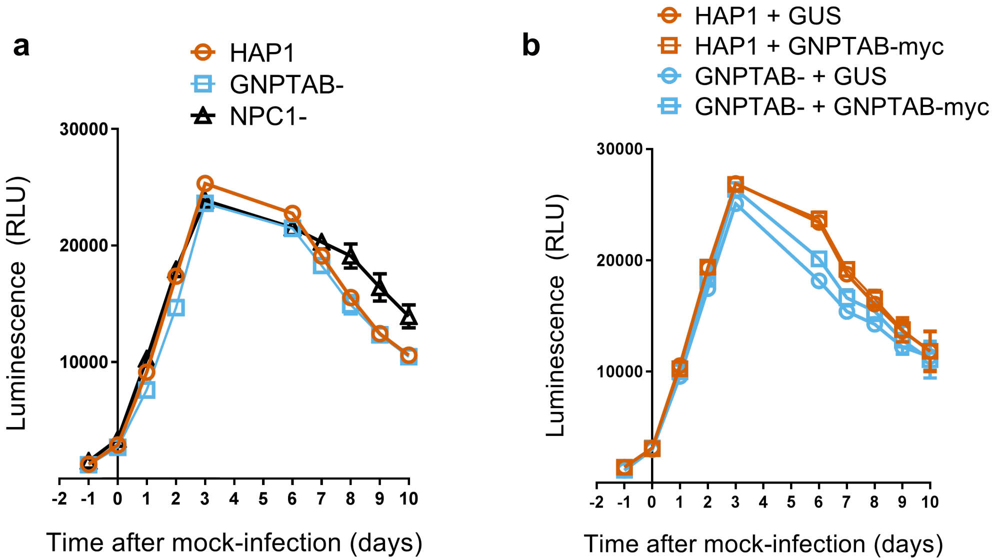

Supplementary Figure 1: *Growth of parental HAP1, GNPTAB- and NPC1- knockout, and reconstituted cells over time.* (a) Parental HAP1 (orange circles), GNPTAB- (blue squares) and NPC1- (black triangles). (b) HAP1 (orange) or GNPTAB- (blue) cells, transduced to express GNPTAB-myc (squares) or GUS (circles). Cells were seeded as for Fig.2b, but were mock-infected, and cell growth was followed over time using CellTiter-Glo. Data represent the mean ± s.d. of 8 biological replicates. A representative of 2 independent experiments is shown.

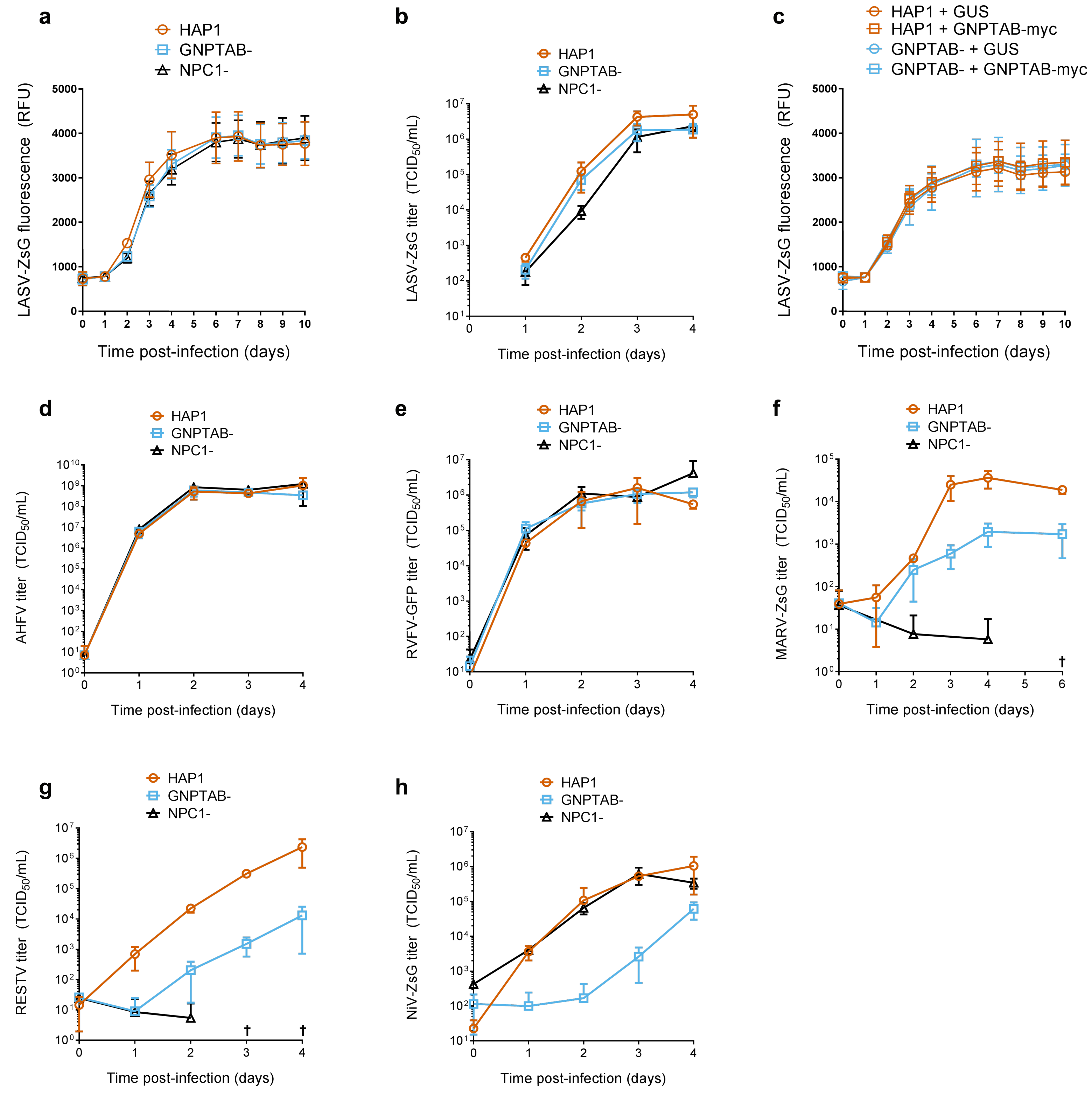

Supplementary Figure 2: *Growth of miscellaneous viruses in parental, knockout and reconstituted cells.* (a) LASV-ZsG fluorescence in infected parental HAP1 (orange circles), GNPTAB- (blue squares) and NPC1- (black triangles) cells. Cells were seeded as for Fig.2b, infected with the reporter LASV-ZsG virus at a MOI of 0.1 and fluorescence was followed over time. Data represent the mean ± s.d. of 4 biological replicates. A representative of two independent experiments is shown. (b) Yields of LASV-ZsG released from infected cells. Cells were seeded and infected as for panel (a). Samples of supernatant were taken over time and LASV-ZsG titers were determined on Vero E6 cells. Data represent the mean of 4 biological replicates ± s.d. (c) LASV-ZsG fluorescence in reconstituted cell-lines; as for panel (a), but using HAP1 (orange) or GNPTAB- (blue) cells, transduced to express GNPTAB-myc (squares) or GUS (circles). Data represent the mean ± s.d. of 8 biological replicates. A representative of 2 independent experiments is shown. (d) Growth of AHFV in HAP1 cells. (e) Growth of RVFV-GFP in HAP1 cells. (f) Growth of MARV-ZsG in HAP1 cells. (g) Growth of RESTV in HAP1 cells. (h) Growth of NiV-ZsG in HAP1 cells. For panels (d) to (h), HAP1 cells were seeded as for Fig.2b, and infected at a MOI of 0.1. Samples of culture supernatant were harvested and virus titers were determined in A549 cells for AHFV or in Vero E6 cells for other viruses. Data represent the mean ± s.d. of at least 3 biological replicates. †, virus not detected in supernatants from NPC1- cells.

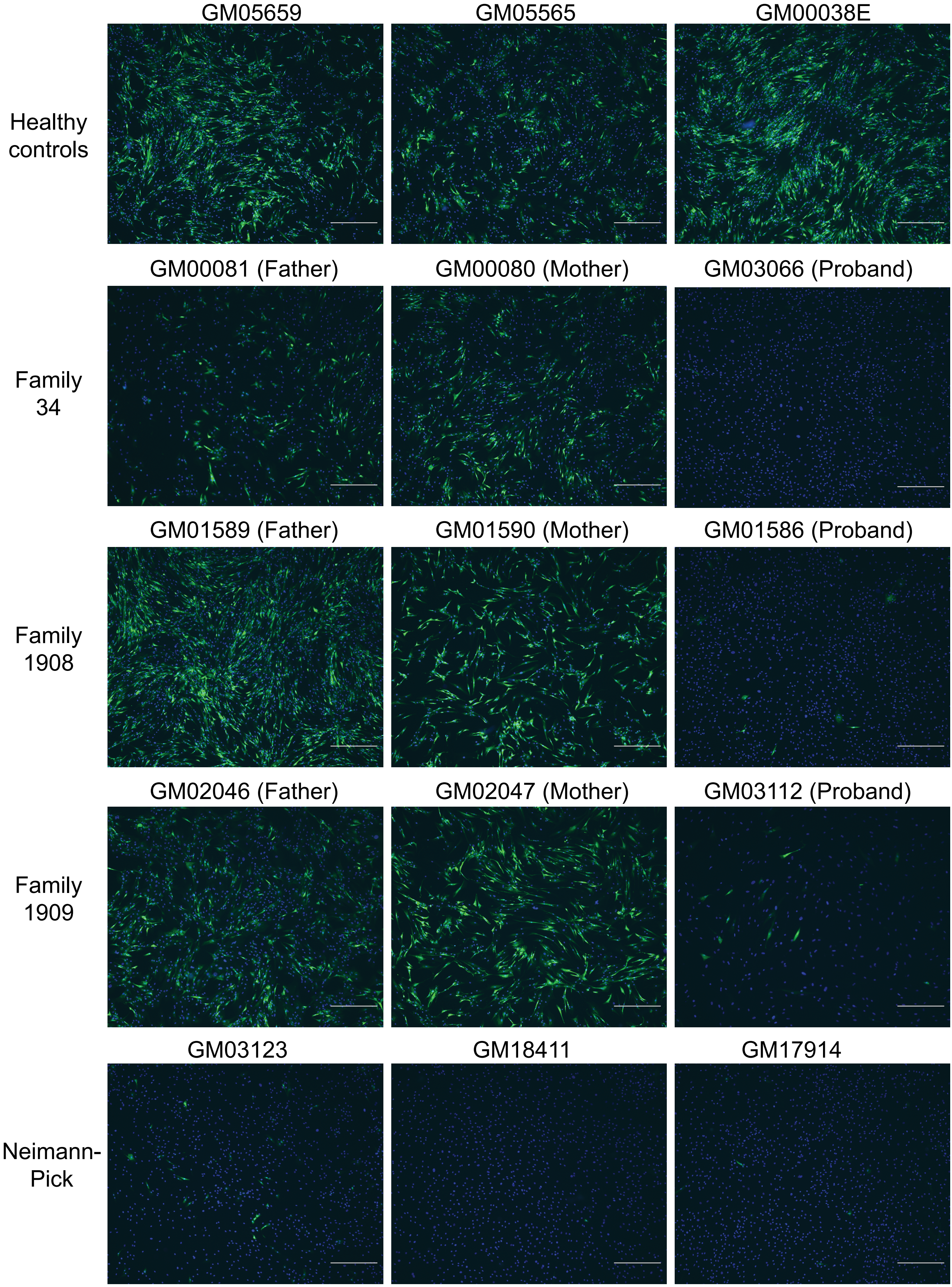

Supplementary Figure 3: *Immunofluorescence of primary human fibroblasts infected with EBOV-ZsG reporter virus.* Cells were seeded at 3000 cells per well of a 96-well plate, and the following day infected with EBOV-ZsG at an MOI of 1. Three days post-infection, the cells were fixed with formalin, permeabilized with PBS supplemented with 0.1% Triton X-100 and nuclei were stained with DAPI. Scale bars represent 100 µm. Micrographs representative of 2 independent experiments are shown.

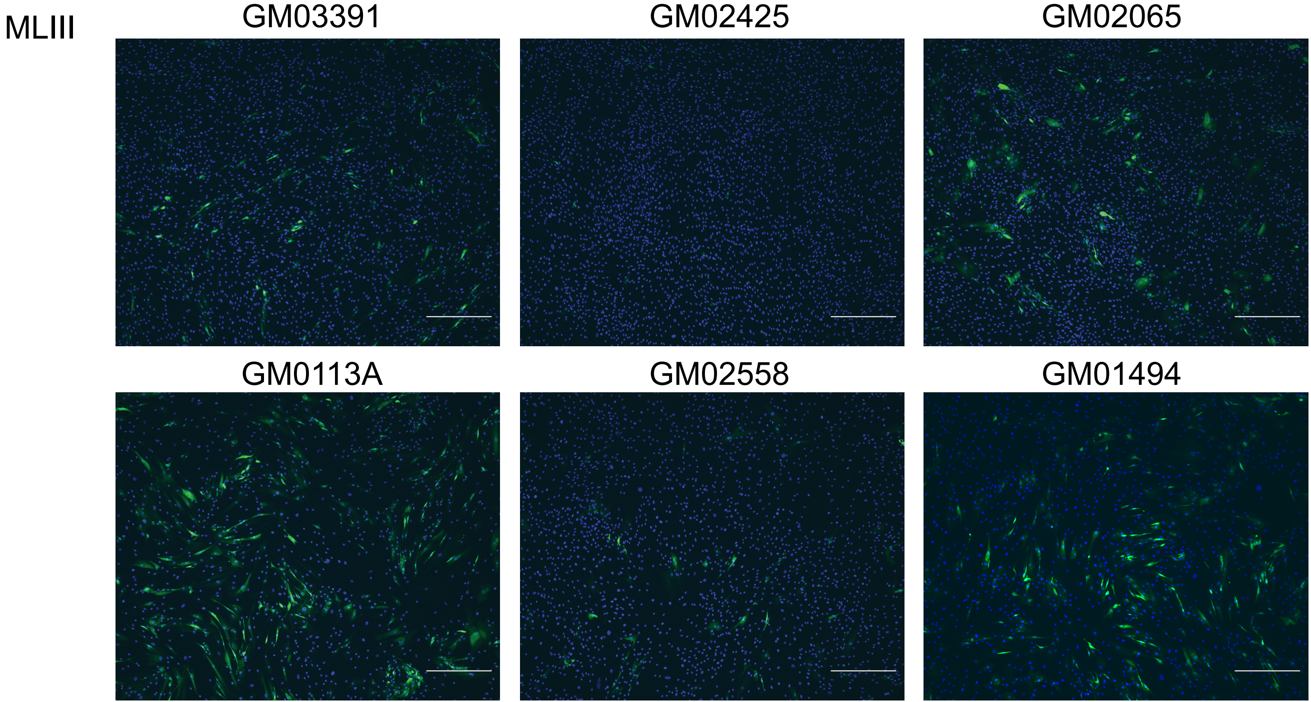

Supplementary Figure 4: *Immunofluorescence of primary fibroblasts from MLIII patients infected with EBOV-ZsG reporter virus.* Cells were seeded, infected and stained as for Supplementary Figure 3. Micrographs representative of 2 independent experiments are shown.

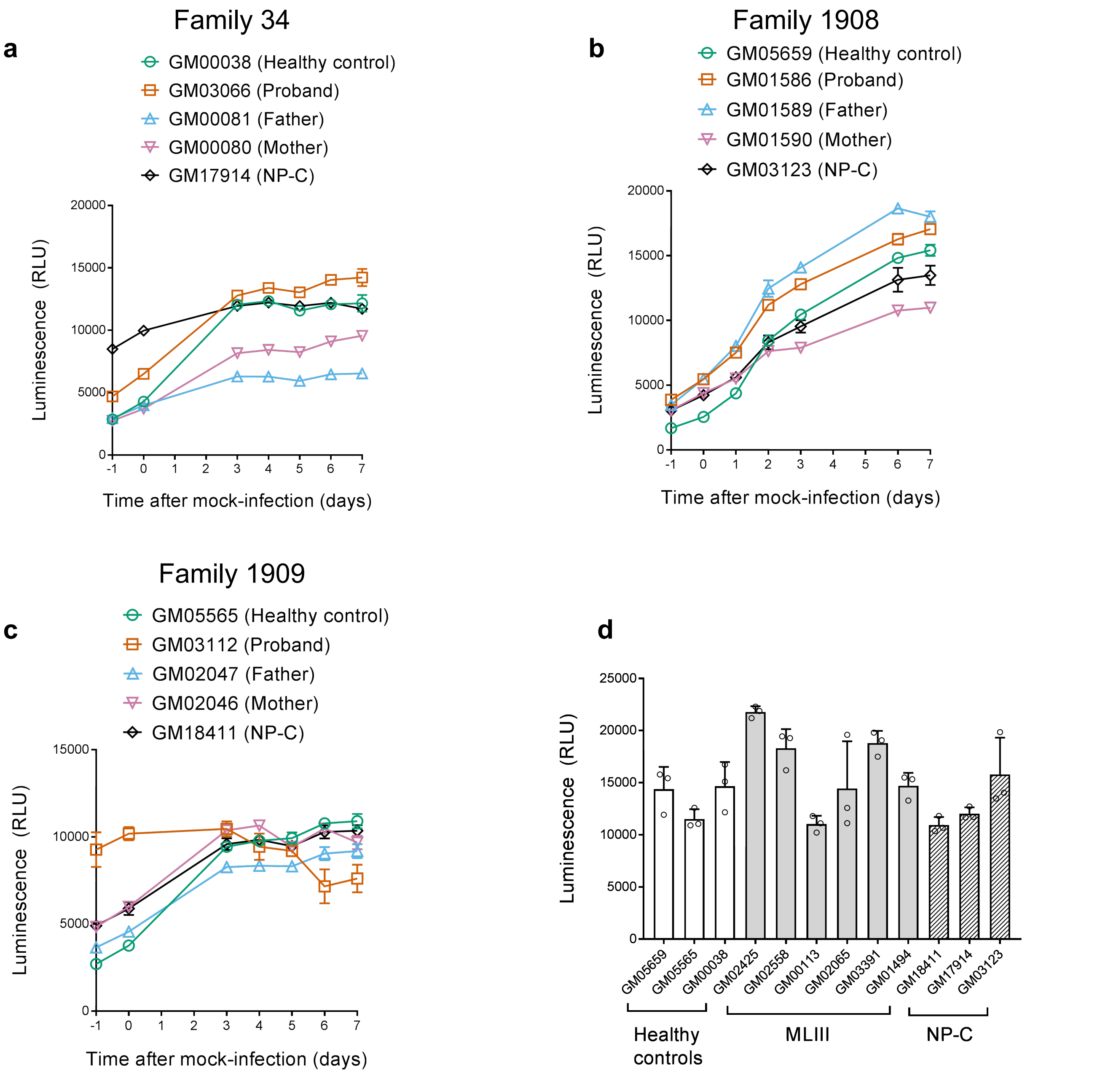

Supplementary Figure 5: *Growth of uninfected primary fibroblasts from mucolipidosis patients over time.* Cells were seeded as for Supplementary Figure 3, but cell viability was determined after various periods using CellTiter-Glo. (a) family 34, (b) family 1908, (c) family 1909. Data represent the mean ± s.d. of 4 biological replicates. One of at least 2 independent experiments is shown. (d) MLIII patients, at day 7 after mock-infection. Cells were seeded and viability determined as for panels a-c. Data represent the mean ± s.d. of 3 independent experiments.

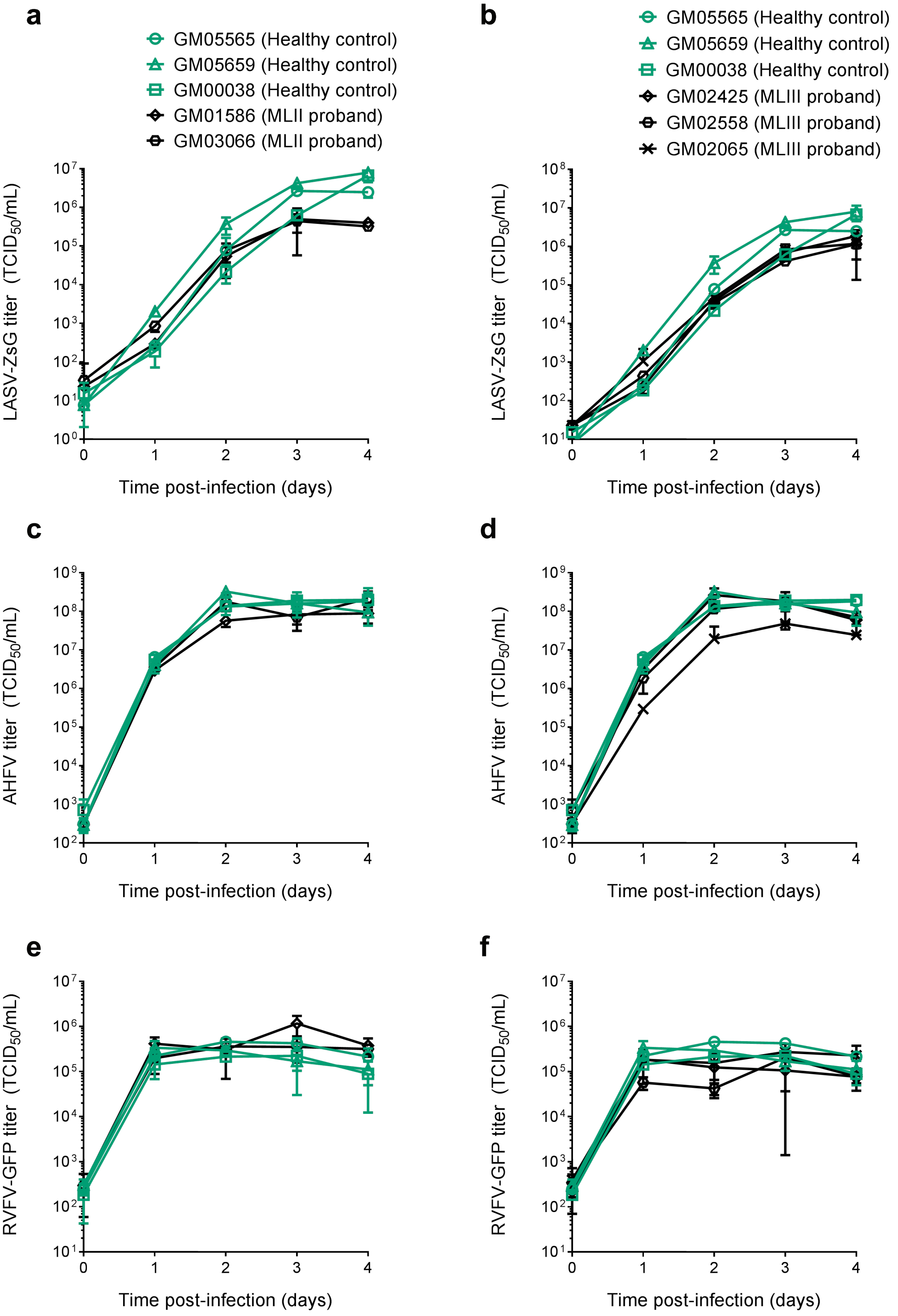

Supplementary Figure 6: *Growth of miscellaneous viruses in primary fibroblasts from healthy controls and mucolipidosis patients over time.* Fibroblast cultures were infected at an MOI of 0.1, samples of culture medium were harvested over time and virus titers determined. (a) LASV-ZsG growth in MLII fibroblasts , (b) LASV-ZsG in MLIII fibroblasts, (c) AHFV in MLII fibroblasts, (d) AHFV in MLIII fibroblasts, (e) RVFV-GFP in MLII fibroblasts, (f) RVFV-GFP in MLIII fibroblasts. Data represent the mean ± s.d. of 3 technical replicates.

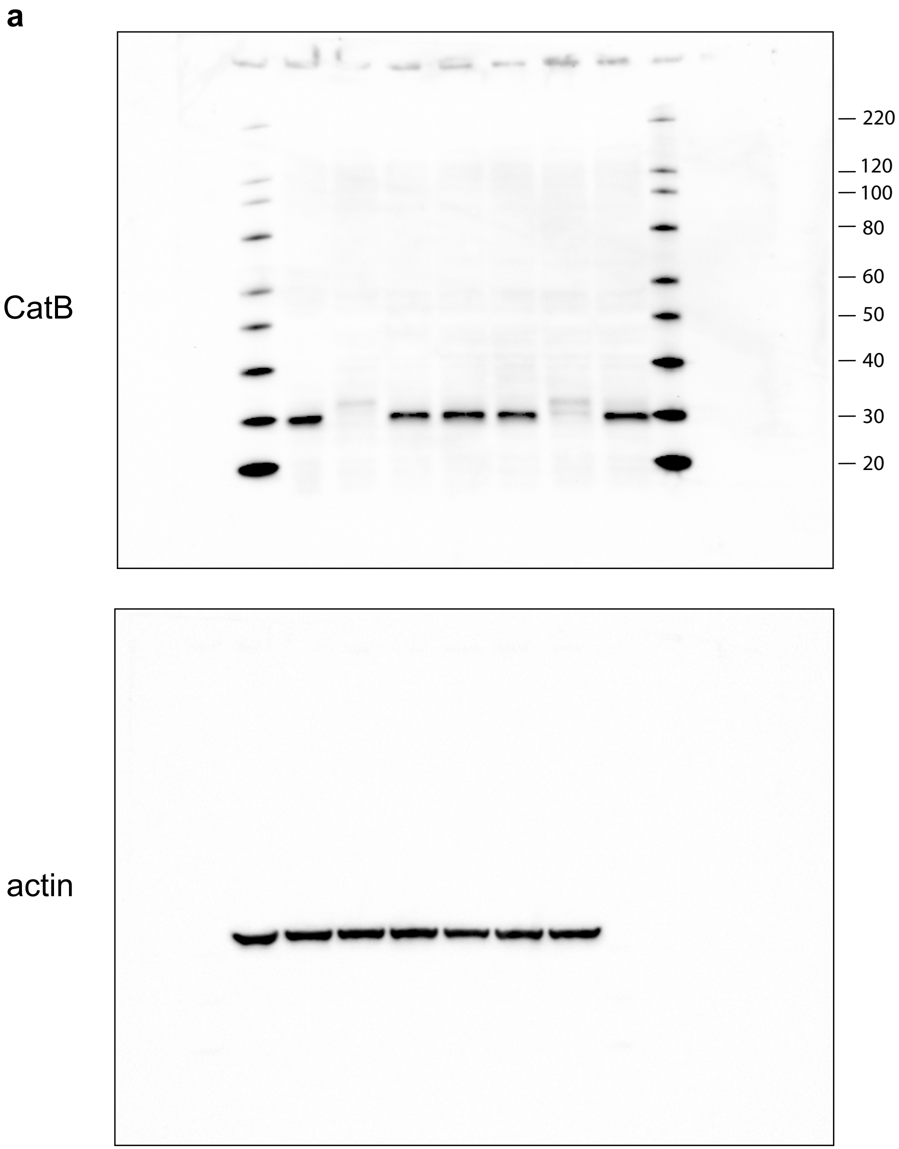

Supplementary Figure 7: *Full immunoblots for the blots shown in Fig.5*. (a) CatB and (b) CatL. The molecular masses of markers are indicated to the right in kDa. CatB and CatL are initially made as procathepsin precursors (~40 kDa). These become activated following proteolytic processing and dissociation of the pro-region at low pH in lysosomes, yielding a single chain of ~30 kDa. Additional proteolytic events can convert the single polypeptide into heavy (27 and 24 kDa) and light (5 kDa) chains, with the loss of a dipeptide. These latter cleavages do not affect the catalytic activity of the enzyme. Oligosaccharides are also trimmed. The major bands detected by both the CatB and CatL antibodies are consistent with the predicted molecular mass of the active single chain forms. Additional bands were not investigated further, but faint bands migrating more slowly than the single-chain cathepsins may represent incompletely processed versions. The band detected by the CatL antibody that migrated around 40 kDa, may be procathepsin L.

b

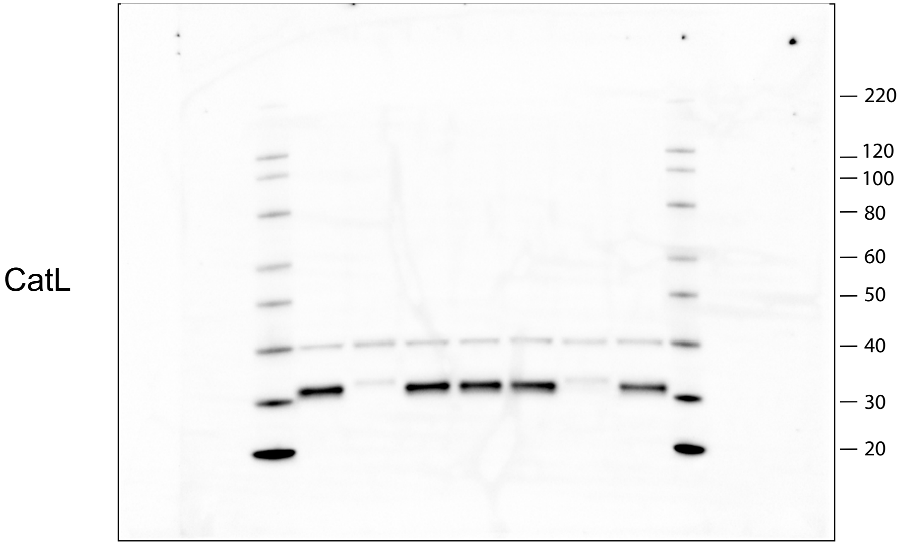

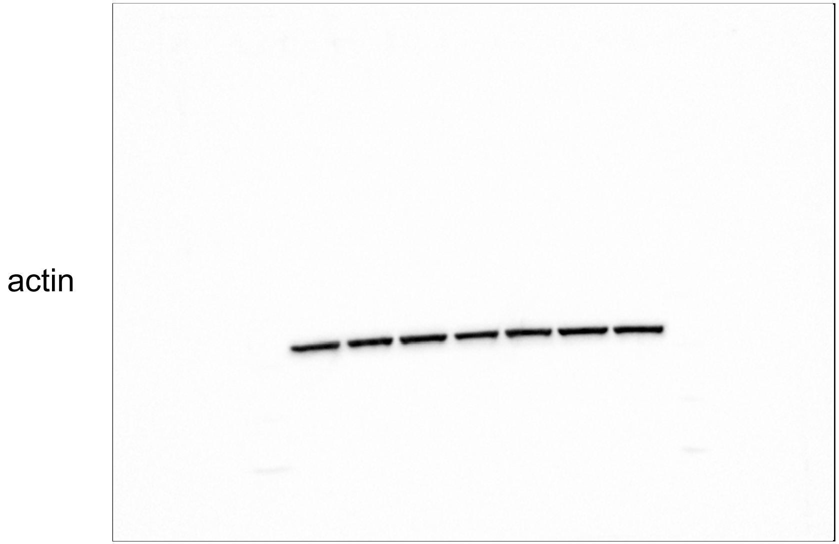

Supplementary Figure 7b

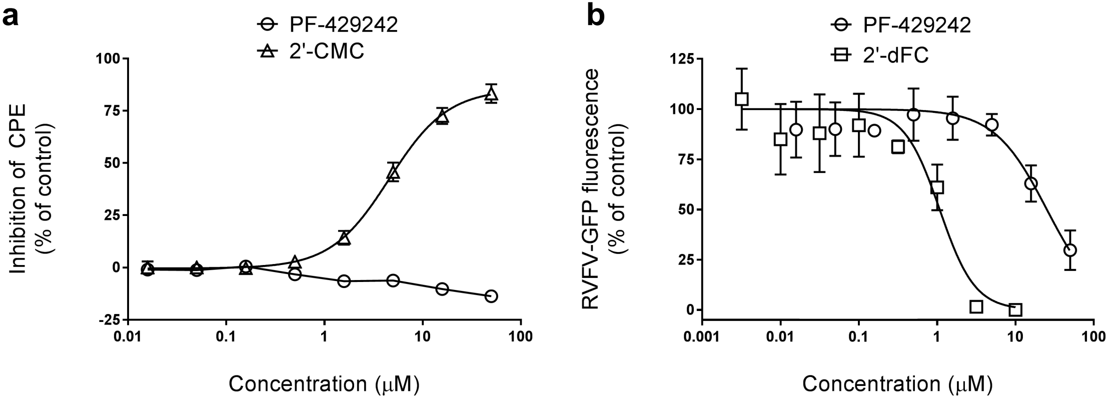

Supplementary Figure 8: *PF-429242 does not block AHFV or RVFV infection*. (a) A549 cells were treated with PF-429242, or control compound 2'-CMC, for 2 hours prior to infection with AHFV at an MOI of 0.3. Three days later, cell viability was determined. (b) Huh7 cells were treated with PF-429242, or control compound 2'-dFC, and infected with RVFV-GFP reporter virus at an MOI of 0.05. Three days later, GFP fluorescence was measured. Data represent the mean ± s.d. of 4 biological replicates.

Supplementary Table 1: *Oligonucleotides used in this study.*

PCR, cloning and sequencing primers		
Primer name	Application	5’ to 3’ sequence
NPC1-fwd	Amplify NPC1 region of interest	AACAACTCTTATTTCCTGGCCAATG
NPC1-rev	Amplify NPC1 region of interest	CTGGCCCTATTATGTGTGAGATCAT
NPC1-seq	Sequence NPC1 region of interest	CTGGCCCTATTATGTGTGAGATCAT
GNPTAB-fwd	Amplify GNPTAB region of interest	AACTCAGAAAGACCCCTTAAACTGT
GNPTAB-rev-seq	Amplify and sequence GNPTAB region of interest	TGTCCTTTTCAGGAACTGTAGCTTA
GNPTAB-att-fwd	Amplify GNPTAB for cloning	GGGGACAAGTTTGTACAAAAAAGCAGGCTATGGATCCACTAGTCCAGTGTGG
GNPTAB-myc-att-rev	Amplify GNPTAB, introduce myc-tag for cloning	GGGGACCACTTTGTACAAGAAAGCTGGGTATCACTACAGATCCTCTTCTGAGATGAGTTT

CRISPR sequencing primers	
Primer	5’ to 3’ sequence
PCR#1-Forward	AATGGACTATCATATGCTTACCGTAACTTGAAAGTATTTCG
PCR#2-Reverse	TGTGGGCGATGTGCGCTCTG
F01	AATGATACGGCGACCACCGAGATCTACACTCTTTCCCTACACGACGCTCTTCCGATCTtAAGTAGAGtcttgtggaaaggacgaaacaccg
F02	AATGATACGGCGACCACCGAGATCTACACTCTTTCCCTACACGACGCTCTTCCGATCTatACACGATCtcttgtggaaaggacgaaacaccg
F03	AATGATACGGCGACCACCGAGATCTACACTCTTTCCCTACACGACGCTCTTCCGATCTgatCGCGCGGTtcttgtggaaaggacgaaacaccg
F04	AATGATACGGCGACCACCGAGATCTACACTCTTTCCCTACACGACGCTCTTCCGATCTcgatCATGATCGtcttgtggaaaggacgaaacaccg
F05	AATGATACGGCGACCACCGAGATCTACACTCTTTCCCTACACGACGCTCTTCCGATCTtcgatCGTTACCAtcttgtggaaaggacgaaacaccg
F06	AATGATACGGCGACCACCGAGATCTACACTCTTTCCCTACACGACGCTCTTCCGATCTatcgatTCCTTGGTtcttgtggaaaggacgaaacaccg
R01	CAAGCAGAAGACGGCATACGAGATTCGCCTTAGTGACTGGAGTTCAGACGTGTGCTCTTCCGATCTATAACGGACTAGCCTTATTTTAAC
R02	CAAGCAGAAGACGGCATACGAGATCTAGTACGGTGACTGGAGTTCAGACGTGTGCTCTTCCGATCTATAACGGACTAGCCTTATTTTAAC
R03	CAAGCAGAAGACGGCATACGAGATTTCTGCCTGTGACTGGAGTTCAGACGTGTGCTCTTCCGATCTATAACGGACTAGCCTTATTTTAAC
R04	CAAGCAGAAGACGGCATACGAGATGCTCAGGAGTGACTGGAGTTCAGACGTGTGCTCTTCCGATCTATAACGGACTAGCCTTATTTTAAC
