# Supplementary material for: A genome-wide CRISPR screen identifies N-acetylglucosamine-1-phosphate transferase as a potential antiviral target for Ebola virus
Source: Nat Commun. 2019 Jan 17;10:285. doi: 10.1038/s41467-018-08135-4 (PMC6336797; doi:10.1038/s41467-018-08135-4)
Supplement: Supplementary file 3 — Description of Additional Supplementary Files [file 41467_2018_8135_MOESM3_ESM.docx]

**Description of Additional Supplementary Files**

**File Name:** Supplementary Data 1

**Description:** MAGeCK analysis output of CRISPR screen at the sgRNA level.

**File Name:** Supplementary Data 2

**Description:** MAGeCK analysis output of CRISPR screen at the gene level.
