# Supplementary material for: A genome-wide CRISPR screen identifies N-acetylglucosamine-1-phosphate transferase as a potential antiviral target for Ebola virus
Source: Nat Commun. 2019 Jan 17;10:285. doi: 10.1038/s41467-018-08135-4 (PMC6336797; doi:10.1038/s41467-018-08135-4)
Supplement: Supplementary file 6 — Reporting summary [file 41467_2018_8135_MOESM6_ESM.pdf]

nature research

Corresponding author(s): *Double-blind peer review submissions: write DBPR and your manuscript number here instead of author names.*

Reporting Summary

Nature Research wishes to improve the reproducibility of the work that we publish. This form provides structure for consistency and transparency in reporting. For further information on Nature Research policies, see Authors & Referees and the Editorial Policy Checklist.

Statistical parameters

When statistical analyses are reported, confirm that the following items are present in the relevant location (e.g. figure legend, table legend, main text, or Methods section).

n/a	Confirmed	
☐	☒	The exact sample size (n) for each experimental group/condition, given as a discrete number and unit of measurement
☐	☒	An indication of whether measurements were taken from distinct samples or whether the same sample was measured repeatedly
☐	☒	The statistical test(s) used AND whether they are one- or two-sided<br>*Only common tests should be described solely by name; describe more complex techniques in the Methods section.*
☒	☐	A description of all covariates tested
☒	☐	A description of any assumptions or corrections, such as tests of normality and adjustment for multiple comparisons
☐	☒	A full description of the statistics including central tendency (e.g. means) or other basic estimates (e.g. regression coefficient) AND variation (e.g. standard deviation) or associated estimates of uncertainty (e.g. confidence intervals)
☐	☒	For null hypothesis testing, the test statistic (e.g. F, t, r) with confidence intervals, effect sizes, degrees of freedom and P value noted<br>*Give P values as exact values whenever suitable.*
☒	☐	For Bayesian analysis, information on the choice of priors and Markov chain Monte Carlo settings
☒	☐	For hierarchical and complex designs, identification of the appropriate level for tests and full reporting of outcomes
☒	☐	Estimates of effect sizes (e.g. Cohen's d, Pearson's r), indicating how they were calculated
☐	☒	Clearly defined error bars<br>*State explicitly what error bars represent (e.g. SD, SE, CI)*

Our web collection on statistics for biologists may be useful.

Software and code

Policy information about availability of computer code

Data collection	Gen5 v2.07 (BioTek) used for data collection on Synergy plate reader, NIS-Elements Basic Research v3.2 (Nikon) used to capture immunofluorescence images, Image Lab v3.2.1 (Bio-Rad) used to capture Immunoblots, HiSeq Control Software (Illumina) for sequencing on HiSeq platform.
Data analysis	CLC Genomics 10 (Qiagen) was used to trim and demultiplex NGS data, MAGeCK software v0.5.7 was used for analysis of the sgRNA sequencing data, Prism 7.0 (GraphPad) was used for plotting graphs and for statistical analysis.

For manuscripts utilizing custom algorithms or software that are central to the research but not yet described in published literature, software must be made available to editors/reviewers upon request. We strongly encourage code deposition in a community repository (e.g. GitHub). See the Nature Research guidelines for submitting code & software for further information.

Data

Policy information about availability of data

All manuscripts must include a data availability statement. This statement should provide the following information, where applicable:

- Accession codes, unique identifiers, or web links for publicly available datasets
- A list of figures that have associated raw data
- A description of any restrictions on data availability

All relevant data are available from the corresponding authors upon request.

Field-specific reporting

Please select the best fit for your research. If you are not sure, read the appropriate sections before making your selection.

☒ Life sciences ☐ Behavioural & social sciences ☐ Ecological, evolutionary & environmental sciences

For a reference copy of the document with all sections, see nature.com/authors/policies/ReportingSummary-flat.pdf

Life sciences study design

All studies must disclose on these points even when the disclosure is negative.

Sample size	Sample sizes were based on standards in the field, typically 3 independent biological replicates, with each replicate assayed in technical duplicate or triplicate.
Data exclusions	No data were excluded from the analysis.
Replication	Yes, for example, GNPTAB was a hit in the CRISPR screen for genes important for Ebola infection - this was then validated as infection was impaired in GNPTAB-knockout HAP1 cells, but was restored upon reconstitution of GNPTAB expression.
Randomization	Fibroblasts from patient families were studied together, i.e. fibroblasts from father, mother and proband (along with healthy control and NP-C patient cells) were tested back-to-back.
Blinding	Blinding not relevant to this study.

Reporting for specific materials, systems and methods

Materials & experimental systems

n/a	Involved in the study	
☐	☒	Unique biological materials
☐	☒	Antibodies
☐	☒	Eukaryotic cell lines
☒	☐	Palaeontology
☒	☐	Animals and other organisms
☒	☐	Human research participants

Methods

n/a	Involved in the study	
☒	☐	ChIP-seq
☒	☐	Flow cytometry
☒	☐	MRI-based neuroimaging

Unique biological materials

Policy information about availability of materials

Obtaining unique materials	Patient fibroblasts are available from the Coriell Institute, HAP1 knockout cells are available from Horizon Genomics.

Antibodies

Antibodies used	Anti-GNPTAB (#PA5-69636, ThermoFisher), anti-myc (clone 9E10, #MA1-980, ThermoFisher), anti-NPC1 (#108921, Abcam), anti-actin (clone AC-15, #A5441, Sigma-Aldrich), anti-CatB (#31718, Cell Signaling Technology) and anti-CatL (#AF952, R&D Systems).
Validation	Anti-GNPTAB and anti-myc, detect bands of expected size upon GNPTAB-myc expression in knockout cells, but not with a control

construct (Fig. 2). Anti-NPC1, lack of detection of a band of expected size in knockout cells (Fig. 2 and manufacturer's website). Anti-CatB, absence of band correlates with lack of activity in biochemical peptide cleavage assay (Fig. 5).

Eukaryotic cell lines

Policy information about cell lines

Cell line source(s)	Fibroblast cell lines were from Coriell Institute. Huh7 cells were from Apath, LLC. Huh7.5.1 cells were from Francis Chisari. A549 and Vero E6 cells were from the CDC core facility.
Authentication	None of the cell lines were formally authenticated in our laboratory.
Mycoplasma contamination	All cell lines were negative for mycoplasma.
Commonly misidentified lines (See ICLAC register)	No commonly misidentified cell lines were used in this study.
